# Quality of Life During and After Completion of Neoadjuvant Chemoradiotherapy for Esophageal and Junctional Cancer

**DOI:** 10.1245/s10434-019-07779-w

**Published:** 2019-10-16

**Authors:** B. J. Noordman, M. G. E. Verdam, B. Onstenk, J. Heisterkamp, W. J. B. M. Jansen, I. S. Martijnse, S. M. Lagarde, B. P. L. Wijnhoven, C. M. M. Acosta, A. van der Gaast, M. A. G. Sprangers, J. J. B. van Lanschot

**Affiliations:** 1 Department of Surgery, Erasmus MC – University Medical Centre Rotterdam, Rotterdam, The Netherlands; 2 Department of Medical Psychology, Academic Medical Centre, Amsterdam, The Netherlands; 3 Department of Surgery, Elisabeth-Tweesteden Hospital, Tilburg, The Netherlands; 4 Department of Medical Oncology, Erasmus MC – University Medical Centre Rotterdam, Rotterdam, The Netherlands

## Abstract

**Background:**

The course of health-related quality of life (HRQOL) during and after completion of neoadjuvant chemoradiotherapy (nCRT) for esophageal or junctional carcinoma is unknown.

**Methods:**

This study was a multicenter prospective cohort investigation. Patients with esophageal or cancer to be treated with nCRT plus esophagectomy were eligible for inclusion in the study. The HRQOL of the patients was measured with European Organization for Research and Treatment of Cancer QLQ-C30, QLQ-OG25, and QLQ-CIPN20 questionnaires before and during nCRT, then 2, 4, 6, 8, 10, 12, 14, and 16 weeks after nCRT and before surgery. Predefined end points were based on the hypothesized impact of nCRT. The primary end points were physical functioning, odynophagia, and sensory symptoms. The secondary end points were global quality of life, fatigue, weight loss, and motor symptoms. Mixed modeling analysis was used to evaluate changes over time.

**Results:**

Of 106 eligible patients, 96 (91%) were included in the study. The rate of questionnaires returned ranged from 94% to 99% until week 12, then dropped to 78% in week 16 after nCRT. A negative impact of nCRT on all HRQOL end points was observed during the last cycle of nCRT (all *p* < 0.001) and 2 weeks after nCRT (all *p* < 0.001). Physical functioning, odynophagia, and sensory symptoms were restored to pretreatment levels respectively 8, 4, and 6 weeks after nCRT. The secondary end points were restored to baseline levels 4–6 weeks after nCRT. Odynophagia, fatigue, and weight loss improved after nCRT compared with baseline levels at respectively 6 (*p* < 0.001), 16 (*p* = 0.001), and 12 weeks (*p* < 0.001).

**Conclusion:**

After completion of nCRT for esophageal cancer, HRQOL decreases significantly, but all HRQOL end points are restored to baseline levels within 8 weeks. Odynophagia, fatigue, and weight loss improved 6–16 weeks after nCRT compared with baseline levels.

**Electronic supplementary material:**

The online version of this article (10.1245/s10434-019-07779-w) contains supplementary material, which is available to authorized users.

Neoadjuvant chemoradiotherapy (nCRT) followed by surgery is a standard of care for patients with potentially curable esophageal or esophagogastric junctional cancer.^1^,^2^ Although esophagectomy has a profound impact on both long- and short-term patient health-related quality of life (HRQOL), addition of nCRT to surgery does not jeopardize HRQOL after surgery compared with surgery alone.^3^,^4^ However, immediately after completion of nCRT (before surgery), patients show a profound drop in HRQOL compared with baseline levels.^3^,^5^,^6^ This deterioration improves after surgery, suggesting that HRQOL is restored in the period between completion of nCRT and surgery.^3^,^5^ However, the detailed course of HRQOL during and after completion of nCRT is unknown. Such information might have an impact on the timing of surgery, although this is debated. Traditionally, surgery is scheduled 4–6 weeks after completion of nCRT. However, findings have shown that a longer time to surgery (up to 12 weeks) does not endanger oncologic outcome.^7^ Increasing the time to surgery allows patients to recover from nCRT and optimize their physical condition before surgery. Furthermore, a longer waiting time to surgery is suggested to increase the pathologically complete response rate (i.e., no viable tumor cells in the resection specimen), which might improve prognostication.^7^

This study aimed primarily to assess the course of HRQOL in the period from the start of nCRT until surgery for patients with locally advanced esophageal or junctional carcinoma.

## Methods

A multicenter prospective cohort study was conducted. Patients with locally advanced esophageal or esophago-gastric junctional cancer, as determined by endoscopic ultrasound, computed tomography (CT) and/or positron emission tomography (PET)-CT, who would be undergoing nCRT according to the ChemoRadiotherapy for Oesophageal cancer followed by Surgery Study (CROSS) regimen (weekly administration of carboplatin and paclitaxel plus 41.4-Gy concurrent radiotherapy) were considered eligible for the study.^1^ Patients considered insufficiently fluent in the Dutch language or cognitively unable to understand the questionnaire were excluded. Consecutive patients were recruited before the start of nCRT in the Erasmus MC–University Medical Centre, Rotterdam, and in the Elisabeth-Tweesteden Hospital, Tilburg. The study was approved by the ethics committee of the Erasmus MC (MEC-2016-250).

### HRQOL Measurement

Patients were informed about the study by their own physician. Subsequently, patients were asked to participate via telephone by one of the investigators. Participating patients received the self-report questionnaires by mail and were asked by telephone to complete the questionnaire at baseline (before nCRT), at the date of the last nCRT cycle, and every 2 weeks thereafter until the date of surgery, with a maximum follow-up period of 16 weeks after completion of nCRT. All the patients completed the questionnaires themselves and were reminded twice via telephone by one of the investigators during each assessment.

Cancer-related general HRQOL was measured with the European Organization for Research and Treatment of Cancer (EORTC) QLQ-C30, a validated questionnaire for cancer patients.^8^ Esophageal cancer-specific HRQOL was assessed with the EORTC QLQ-OG25, a validated questionnaire for patients with cancer of the esophagus, the esophago-gastric junction, and the stomach.^9^ Chemotherapy-induced peripheral neuropathy (CIPN) symptoms were assessed using the EORTC QLQ-CIPN20, a questionnaire designed to elicit patients’ experience of symptoms related to CIPN.^10^

Before the start of the study, end points were defined by individual consensus discussion with upper gastrointestinal (GI) surgical oncologists, medical oncologists, and nurse practitioners. One primary end point and one or two secondary end points from each questionnaire were chosen based on the hypothesized impact of nCRT. This led to assignment of physical functioning (EORTC QLQ-C30), odynophagia (EORTC QLQ-OG-25), and sensory symptoms (EORTC QLQ-CIPN20) as primary end points, and to global quality of life, fatigue (both QLQ-C30), weight loss (EORTC QLQ-OG25), and motor symptoms (EORTC QLQ-CIPN20) as secondary end points.

### Statistical Analysis

Data were analyzed on an intention-to-treat basis. Pretreatment clinicopathologic characteristics were collected and described. Questionnaire scores were transformed into a 0–100 scale according to EORTC guidelines.^11^ Higher scores for functional and global scales (e.g., physical functioning and global quality of life) indicate better HRQOL. Higher scores on symptom scales (e.g., fatigue) indicate worse HRQOL.

Over-time changes in the follow-up measurements were analyzed using mixed modeling analysis, a technique that enables analysis of all completed questionnaires by allowing for inclusion of data from patients with different numbers of completed measurements.^12^ Mean over-time differences were described.

Cohen’s d (CD) effect sizes based on the beta estimates from the mixed modeling analyses were used to allow for standardized comparison between different end points and to assess clinical relevance of the found effects. The CD values of 0.2, 0.5, and 0.8 indicate small, medium, and large effects, respectively.^13^ Effect sizes of 0.5 or larger were defined as clinically relevant.^14^

On an exploratory basis, we investigated the effects of several background variables on the trajectory of HRQOL scores. Because the investigated sample showed variation in timing of surgery, this could have influenced the course of HRQOL.

Some patients (*n* = 29) participated in the diagnostic preSANO trial.^15^,^16^ In that trial, the patients underwent a clinical response evaluation (CRE) using endoscopy with biopsies, endoscopic ultrasound, and PET-CT 4–6 weeks after nCRT to determine the accuracy of residual disease detection. The patients with residual disease or non-passable tumor during the clinical response evaluation after 4–6 weeks underwent immediate surgical resection, whereas the remaining patients had surgery 10–14 weeks after completion of nCRT.

Patients with (substantial) residual disease after nCRT might experience worse HRQOL after nCRT, which potentially induces a bias. Furthermore, variations in time to surgery can be attributed to patient-related characteristics such as comorbidities or general condition. More vulnerable patients could have a longer time until surgery intentionally. This might negatively influence HRQOL at longer follow-up measurements, so HRQOL may improve more strongly at the later measures if all patients could have been included. Therefore, the study included the presence of residual disease during clinical response evaluation (only for patients who participated in the preSANO trial), comorbidities (Charlson Comorbidity Index), the American Society of Anesthesiology (ASA) score, age, gender, histology, and cT stage in a separate analysis to investigate their potential effect on the course of HRQOL.^17^

As a correction for multiple comparisons, a *p* value lower than 0.006 was considered statistically significant (a Bonferroni correction of 0.05/9 was applied because the main analyses included nine comparisons with pretreatment levels). All *p* values were two-sided. Data were analyzed using SPSS version 24.0 (IBM, Chicago, IL, USA).

## Results

Of 106 eligible patients, 96 (91%) were included from May 2016 through June 2017 (10 patients refused participation). The rates of response to the questionnaires ranged from 78 to 99% (Table 1). The median age of the patients was 68 years (interquartile range [IQR], 61–71 years), and 77 (80%) of the patients were men. Most of the patients had cT3 tumor (80%) and suspicious regional lymph nodes (66%; Table 2).

**Table 1:** Eligibility status of the study patients

Status	Baseline	Last cycle	2 Weeks	4 Weeks	6 Weeks	8 Weeks	10 Weeks	12 Weeks	14 Weeks	16 Weeks
Eligible	96	96	96	96	93	88	56	49	42	32
Total returned questionnaires (% of eligible)	95 (99)	90 (94)	93 (97)	92 (96)	89 (96)	83 (94)	51 (91)	46 (94)	37 (88)	25 (78)
Surgery (cumulative)	0	0	0	0	3	8	39	46	53	63
Deceased	0	0	0	0	0	0	1	1	1	1
Too ill	0	4	1	1	1	1	1	1	3	4
Randomly missing/other	1	2	2	3	3	4	4	2	2	3

**Table 2:** Clinicopathologic characteristics of the study patients

Characteristic	(*n* = 96) *n* (%)
Age at inclusion (years)	
Median	68
IQR	61–71
Male sex	77 (80)
Tumor type	
Squamous cell carcinoma	18 (19)
Adenocarcinoma	78 (81)
Clinical T stage^a^	
cT1	1 (1)
cT2	15 (16)
cT3	77 (80)
cT4	3 (3)
Clinical N stage^b^	
cN0	33 (34)
cN1	38 (4)
cN2	19 (20)
cN3	6 (6)
ASA classification^c^	
1	15 (16)
2	65 (68)
3	14 (15)
Missing	2 (2)

*IQR* interquartile range, *ASA* American Society of Anesthesiology

^a^Clinical tumor (cT) stage was assessed via endoscopic ultrasonography or computed tomography (CT) and classified according to the International Union for Cancer Control (IUCC) tumor-node-metastasis (TNM) classification, 7th ed

^b^Clinical lymph-node (*N*) stage was assessed via endoscopic ultrasonography, CT, or 18F-fluorodeoxyglucose positron-emission tomography and classified according to IUCC TNM classification, 7th ed

^c^ASA classification is on a scale of 0 to 5, with lower numbers indicating better physical status, 1 indicating a normal healthy patient, 2 indicating a patient with mild systemic disease, and 3 indicating a patient with severe systemic disease

### Predefined Primary End Points

#### Physical Functioning (Fig. 1a)

The over-time changes in physical functioning levels were statistically significant (*p* < 0.001). Physical functioning had declined at the last cycle of nCRT (− 16; *p* < 0.001; CD − 0.80; 95% confidence interval [CI], − 1.00 to − 0.59) compared with the baseline levels. During the follow-up period, physical functioning improved from 2 to 10 weeks after nCRT (4 vs 2 weeks: + 6; *p* < 0.001; CD 0.30; 95% CI 0.17–0.42; 6 vs 4 weeks: + 6; *p* < 0.001; CD 0.28; 95% CI 0.17–0.39; 8 vs 6 weeks: +5; *p* < 0.001; CD 0.24; 95% CI 0.15–0.32; 10 vs 8 weeks: + 3; *p *= 0.003; CD 0.15; 95% CI 0.05–0.24, respectively). From this point, the improvement was no longer statistically significant. Baseline levels were reached at 8 weeks (*p* = 0.95) but were not exceeded during the follow-up period.

**Fig. 1:**
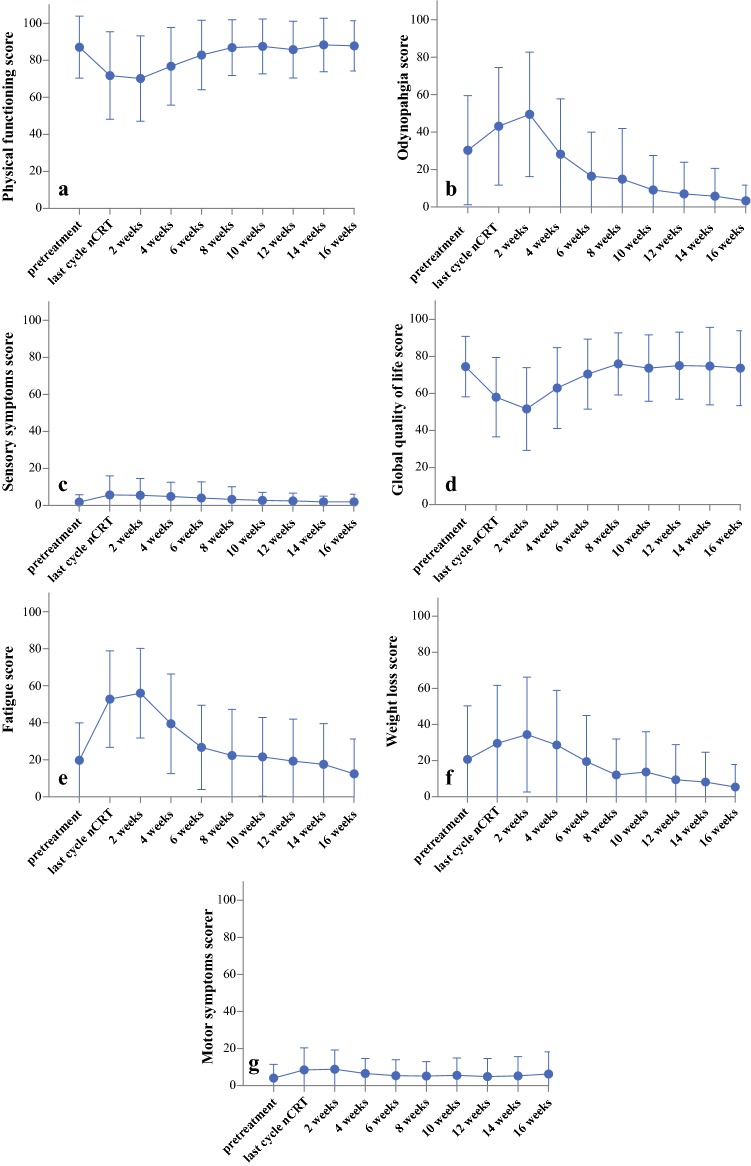
Mean scores with standard deviations for **a** physical functioning, **b** odynophagia, **c** sensory symptoms (primary end points), **d** global quality of life, **e** fatigue, **f** weight loss, and **g** motor symptoms (secondary end points)

#### Odynophagia (Fig. 1b)

The over-time changes in odynophagia levels were statistically significant (*p* < 0.001). Compared with the baseline levels, the odynophagia levels had worsened at the last cycle of nCRT (14; *p* < 0.001; CD − 0.45; 95% CI 0.20–0.70) and remained at that level 2 weeks after nCRT (*p* = 0.038). Thereafter, the odynophagia levels improved from 2 to 4 weeks (− 21; *p* < 0.001; CD − 0.69; 95% CI − 0.89 to − 0.49), and from 4 to 6 weeks (− 11; *p* < 0.001; CD − 0.37; 95% CI − 0.50 to − 0.24). After that, improvement was no longer statistically significant compared with the previous measurement. At 4 weeks after nCRT, baseline levels were reached (*p* = 0.68), and at 6 weeks, the odynophagia levels had improved compared with baseline levels (6 weeks: − 15; *p* < 0.001; CD − 0.42; 95% CI − 0.64 to − 0.20; 10 weeks: − 24; *p* < 0.001; CD − 0.77; 95% CI − 0.98 to − 0.57).

#### Sensory Symptoms (Fig. 1c)

Generally, the over-time changes in sensory symptoms were not statistically significant (*p* = 0.009). However, the specific comparisons between occasions showed that sensory symptoms had worsened at the last cycle of nCRT compared with pretreatment levels, (+4: *p* < 0.001; CD 0.53; 95% CI 0.28–0.80). At 6 weeks after nCRT, sensory symptoms had returned to baseline levels (*p* = 0.013). No further statistically significant improvement compared with previous measurements was observed.

### Predefined Secondary End Points

#### Global Quality of Life (Fig. 1d)

The global quality-of-life scores showed statistically significant changes over time (*p* < 0.001). At the last cycle of nCRT, the global quality-of-life scores had declined (− 16: *p* < 0.001; CD − 0.77; 95% CI − 0.96 to − 0.57) and had further worsened 2 weeks thereafter (− 6: *p* = 0.002; CD − 0.29; 95% CI − 0.47 to − 0.11). From 2 to 8 weeks after nCRT, the global quality-of-life levels improved compared with the previous measurement (4 vs 2 weeks: + 11; *p* < 0.001; CD 0.51; 95% CI 0.33–0.69; 6 vs 4 weeks: + 7; *p* < 0.001; CD 0.34; 95% CI 0.19–0.49; 8 vs 6 weeks: + 5; *p* = 0.001; CD 0.24; 95% CI 0.10–0.39, respectively). After that, improvement was no longer statistically significant. At 6 weeks after nCRT, baseline levels were reached (*p* = 0.031). The baseline levels were not exceeded during the follow-up period.

#### Fatigue (Fig. 1e)

Over time, the fatigue levels changed significantly (*p* < 0.001). Compared with baseline, the fatigue levels had increased at the last cycle of nCRT (+ 34: *p* < 0.001; CD 1.21; 95% CI 1.04–1.39) and remained stable until 2 weeks after nCRT (*p *= 0.32). After that, improvement was observed until 6 weeks compared with the previous measurements (4 vs 2 weeks: − 15; *p* < 0.001; CD − 0.57; 95% CI − 0.73 to − 0.41; 6 vs 4 weeks: − 13; *p* < 0.001; CD − 0.46; 95% CI − 0.60 to − 0.32). Baseline levels were reached at 6 weeks (*p* = 0.007). Thereafter, no statistically significant improvement compared with the previous measurement was observed. Compared with baseline levels, improvement was observed 16 weeks after nCRT (− 8; *p* = 0.001; CD − 0.28; 95% CI − 0.44 to − 0.11).

#### Weight Loss (Fig. 1f)

The weight loss scores changed significantly over time (*p* < 0.001). At the last cycle of nCRT, weight loss had worsened compared with baseline levels (+ 10: *p* = 0.002; CD 0.36; 95% CI 0.13–0.58) and did not improve 2 and 4 weeks after nCRT compared with the previous measurement (*p* = 0.263 and 0.038, respectively). The scores then returned to baseline levels 4 weeks after nCRT (*p* = 0.031) and improved further (6 vs 4 weeks: 9; *p* < 0.001; CD − 0.31; 95% CI − 0.47 to − 0.16; 8 vs 6 weeks: − 7; *p* < 0.001; CD − 0.24; 95% CI − 0.37 to − 0.12). At 12 weeks after nCRT, the weight loss scores had improved compared with baseline levels (− 15; *p* < 0.001; CD − 0.52; 95% CI − 0.79 to − 0.26).

#### Motor Symptoms (Fig. 1g)

The over-time change in motor symptoms was statistically significant (*p* < 0.001). Motor symptoms had worsened at the last cycle of nCRT (+  4; *p* < 0.001). At 4 weeks after nCRT, the motor symptoms had returned to baseline levels (*p* = 0.028). No further improvements in motor symptoms compared with previous measurements was observed.

#### Other End Points

The mean scores of HRQOL domains, except for the predefined end points, are presented in Table 3.

**Table 3:** Mean scores for all domains in the three European Organization for Research and Treatment of Cancer (EORTC) questionnaires that were not predefined end points

Status	Baseline	Last cycle	2 Weeks	4 Weeks	6 Weeks	8 Weeks	10 Weeks	12 Weeks	14 Weeks	16 Weeks
QLQ-C30										
Functional scales										
Role	82 ± 23	56 ± 32	50 ± 32	26 ± 30	71 ± 27	79 ± 23	81 ± 22	81 ± 25	82 ± 25	85 ± 25
Emotional	75 ± 20	74 ± 24	72 ± 23	78 ± 19	80 ± 20	81 ± 17	81 ± 19	83 ± 16	82 ± 17	82 ± 19
Cognitive	91 ± 16	81 ± 25	82 ± 20	86 ± 19	91 ± 16	92 ± 16	91 ± 17	93 ± 14	93 ± 13	95 ± 12
Social	88 ± 18	70 ± 30	69 ± 27	78 ± 23	86 ± 20	88 ± 18	89 ± 18	89 ± 18	90 ± 19	91 ± 18
Symptom scores										
Nausea and vomiting	12 ± 23	28 ± 30	32 ± 32	13 ± 20	10 ± 19	5 ± 15	3 ± 8	5 ± 11	5 ± 11	1 ± 3
Pain	14 ± 19	32 ± 28	38 ± 31	22 ± 26	14 ± 20	11 ± 20	12 ± 22	11 ± 21	12 ± 24	9 ± 19
Dyspnea	8 ± 16	20 ± 26	22 ± 26	20 ± 26	12 ± 21	11 ± 21	11 ± 21	13 ± 19	11 ± 19	8 ± 15
Insomnia	27 ± 30	35 ± 33	33 ± 34	24 ± 31	20 ± 25	16 ± 24	18 ± 24	17 ± 26	14 ± 20	12 ± 25
Loss of appetite	21 ± 28	46 ± 35	52 ± 35	33 ± 34	18 ± 26	12 ± 24	12 ± 22	12 ± 24	11 ± 21	7 ± 17
Constipation	9 ± 21	25 ± 33	25 ± 32	13 ± 24	7 ± 16	5 ± 14	7 ± 15	4 ± 13	4 ± 10	7 ± 17
Diarrhea	6 ± 17	16 ± 26	15 ± 26	5 ± 16	4 ± 12	5 ± 13	7 ± 13	3 ± 9	4 ± 13	5 ± 12
Financial worries	3 ± 12	8 ± 22	6 ± 18	5 ± 14	5 ± 17	5 ± 16	4 ± 16	4 ± 13	4 ± 10	4 ± 11
QLQ-OG25										
Symptom scores										
Dysphagia	27 ± 25	41 ± 28	56 ± 30	25 ± 25	16 ± 19	13 ± 21	10 ± 17	6 ± 12	6 ± 15	4 ± 7
Eating	42 ± 28	57 ± 28	62 ± 28	40 ± 31	27 ± 28	20 ± 27	16 ± 23	13 ± 20	10 ± 17	9 ± 15
Reflux	9 ± 18	14 ± 23	16 ± 26	8 ± 21	5 ± 14	3 ± 10	3 ± 12	3 ± 11	1 ± 6	1 ± 7
Pain and discomfort	15 ± 23	29 ± 28	30 ± 32	22 ± 28	14 ± 23	13 ± 22	10 ± 1912	7 ± 17	7 ± 17	10 ± 20
Anxiety	52 ± 25	46 ± 26	47 ± 27	43 ± 25	41 ± 27	43 ± 26	42 ± 26	41 ± 22	39 ± 26	36 ± 27
Eating with others	27 ± 33	34 ± 35	36 ± 26	21 ± 30	11 ± 24	7 ± 17	5 ± 12	5 ± 14	4 ± 10	1 ± 7
Dry mouth	13 ± 23	26 ± 28	29 ± 30	17 ± 24	13 ± 20	9 ± 20	12 ± 21	13 ± 27	9 ± 22	7 ± 14
Trouble with taste	18 ± 32	44 ± 37	46 ± 35	32 ± 32	21 ± 27	12 ± 24	10 ± 20	8 ± 20	5 ± 12	7 ± 17
Trouble swallowing saliva	13 ± 27	24 ± 32	24 ± 30	14 ± 26	7 ± 18	5 ± 15	3 ± 11	3 ± 12	2 ± 8	3 ± 9
Choking when swallowing	10 ± 22	9 ± 17	9 ± 18	5 ± 14	3 ± 10	4 ± 13	2 ± 9	4 ± 13	5 ± 14	3 ± 9
Trouble with coughing	26 ± 26	32 ± 28	34 ± 28	28 ± 27	21 ± 23	23 ± 24	19 ± 24	19 ± 25	17 ± 22	16 ± 17
Trouble talking	6 ± 18	10 ± 19	13 ± 24	8 ± 18	3 ± 10	3 ± 11	5 ± 13	4 ± 13	4 ± 10	4 ± 11
Hair loss	10 ± 25	22 ± 29	19 ± 26	21 ± 29	19 ± 31	16 ± 26	14 ± 28	14 ± 31	17 ± 36	5 ± 13
QLQ-CIPN20										
Autonomic scale	11 ± 15	21 ± 19	22 ± 19	18 ± 18	14 ± 16	14 ± 15	14 ± 15	14 ± 16	14 ± 18	14 ± 18

Scores are presented as means ± standard deviations

#### Influence of Residual Disease, Comorbidities, and General Condition

Inclusion of residual disease present during the clinical response evaluation, comorbidities (Charlson Comorbidity Index), ASA score, age, gender, histology, and cT stage as control variables did not have an impact on the reported overall trends in HRQOL trajectories (data not shown). However, the patients who had residual disease during CRE exhibited worse odynaphagia levels. The patients with higher Charlson Comorbidity Index (CCI) experienced more fatigue, and the patients with a higher cT stage had more weight loss (Table S1). Furthermore, the patients who had residual disease during CRE or a higher ASA score had increased weight loss over time (Table S2; Fig. S1).

## Discussion

This prospective cohort study showed a profound negative, short-term impact of nCRT on all HRQOL end points for patients who had esophageal or junctional cancer treated with a multimodality regimen based on carboplatin/paclitaxel combined with 41.4 Gy of concurrent radiotherapy. Subsequently, all primary and secondary HRQOL end points were restored to baseline levels 4–10 weeks after completion of nCRT. The odynophagia, fatigue, and weight loss scores even improved after nCRT compared with baseline levels at 6, 16, and 12 weeks, respectively.

This is the first study to investigate the detailed short-term course of HRQOL after nCRT for esophageal or junctional cancer. A previous study showed a negative impact of nCRT on HRQOL 12 weeks after the start of neoadjuvant treatment, which was restored to baseline levels 3 weeks before surgery.^6^ However, this earlier study used a small sample (*n* = 34), only two measurements after nCRT with respect to the start of nCRT rather than the end of nCRT, and the date of surgery, hampering precise assessment of the HRQOL trajectory after nCRT. A study using the Functional Assessment of Cancer Therapy-Esophageal (FACT-E) demonstrated findings similar to those in the current study in terms of return to baseline before surgery, but included only patients who had neoadjuvant chemotherapy.^18^

The HRQOL analysis in the CROSS trial also showed a profound deterioration 1 week after completion of nCRT compared with baseline scores for all primary and secondary HRQOL end points (physical functioning, global quality of life, fatigue, eating, and emotional functioning). However, this study lacked extra measurements between the end of nCRT and the date of surgery.^3^

The results of the current study underscore the value of sufficient recuperation time between completion of nCRT and esophagectomy to enable patients to undergo surgery in optimal physical condition, potentially improving surgical outcome. Moreover, delayed surgery tends to increase the pathologically complete response rate, potentially improving prognosis.^7^,^19^ We recommend that timing of surgery be guided by the patient’s condition. It is advocated that surgery be postponed to as long as 12 weeks after completion of nCRT, and even longer than that when patients experience persisting adverse events or are in bad general condition, especially in the absence of residual disease.

Previous studies have shown lasting deterioration of HRQOL after multimodality treatment for patients with esophageal cancer.^4^,^20^–^22^ Given the current results, these negative findings likely are attributable to esophagectomy and not to chemoradiotherapy per se. Definitive chemoradiotherapy without esophagectomy circumvents the adverse effects of surgery. However, the long-term oncologic outcome is suggested to be inferior to (nCRT plus) surgery.^23^

An active surveillance strategy after completion of nCRT is a topic of investigation in the ESOSTRATE and Surgery As Needed for Oesophageal cancer (SANO) trials.^15^,^24^ With this novel treatment strategy, patients undergo frequent clinical examinations after completion of nCRT, and esophagectomy is offered only to patients with a histologically proven or highly suspected locoregional regrowth without signs of distant dissemination. This active surveillance strategy might reduce the number of patients who need esophagectomy by 30–40%, reducing the impact of surgery on HRQOL. The results of the current study can be used to inform patients for whom a future active surveillance strategy is considered because the stable HRQOL levels during the last measurements likely reflect the HRQOL levels during active surveillance.

The limitations of the current study included the differences in timing of surgery between patients, which introduced different follow-up periods between patients. Nevertheless, inclusion of the confounders, namely, the presence of residual disease during clinical response evaluation (only patients in the preSANO trial), comorbidities (Charlson Comorbidity Index), and ASA score, did not influence the overall trends in HRQOL trajectories.

Another limitation was the potential effect of response shift or reconceptualization of symptoms during treatment (i.e., what was bad before is the new normal). Unfortunately, this is inevitable in HRQOL studies.

In conclusion, HRQOL decreased substantially after completion of nCRT for esophageal cancer, but was restored to baseline levels for all end points within 10 weeks. Odynophagia, fatigue, and weight loss had improved within 16 weeks after nCRT compared with baseline levels. These results suggest a benefit of delaying surgery, especially for vulnerable patients, and can be used to inform patients for whom a future active surveillance strategy is considered.

## Electronic supplementary material

Below is the link to the electronic supplementary material.

Supplementary material 1 (DOCX 72 kb)

## References

[1] van Hagen P, Hulshof MC, van Lanschot JJ (2012). Preoperative chemoradiotherapy for esophageal or junctional cancer. N Engl J Med..

[2] Shapiro J, van Lanschot JJ, Hulshof MC (2015). Neoadjuvant chemoradiotherapy plus surgery versus surgery alone for oesophageal or junctional cancer (CROSS): long-term results of a randomised controlled trial. Lancet Oncol..

[3] Noordman BJ, Verdam MGE, Lagarde SM, et al. Effect of neoadjuvant chemoradiotherapy on health-related quality of life in esophageal or junctional cancer: results from the randomized CROSS trial. *J Clin Oncol.* 2018;36:268–75. 10.1200/JCO.2017.73.7718 29161204

[4] Noordman BJ, Verdam MGE, Lagarde SM (2018). Impact of neoadjuvant chemoradiotherapy on health-related quality of life in long-term survivors of esophageal or junctional cancer: results from the randomized CROSS trial. Ann Oncol..

[5] van Meerten E, van der Gaast A, Looman CW, Tilanus HW, Muller K, Essink-Bot ML (2008). Quality of life during neoadjuvant treatment and after surgery for resectable esophageal carcinoma. Int J Radiat Oncol Biol Phys..

[6] Blazeby JM, Sanford E, Falk SJ, Alderson D, Donovan JL (2005). Health-related quality of life during neoadjuvant treatment and surgery for localized esophageal carcinoma. Cancer..

[7] Shapiro J, van Hagen P, Lingsma HF (2014). Prolonged time to surgery after neoadjuvant chemoradiotherapy increases histopathological response without affecting survival in patients with esophageal or junctional cancer. Ann Surg..

[8] Aaronson NK, Ahmedzai S, Bergman B (1993). The European Organization for Research and Treatment of Cancer QLQ-C30: a quality-of-life instrument for use in international clinical trials in oncology. J Natl Cancer Inst..

[9] Lagergren P, Fayers P, Conroy T (2007). Clinical and psychometric validation of a questionnaire module, the EORTC QLQ-OG25, to assess health-related quality of life in patients with cancer of the oesophagus, the oesophago-gastric junction, and the stomach. Eur J Cancer..

[10] Postma TJ, Aaronson NK, Heimans JJ (2005). The development of an EORTC quality-of-life questionnaire to assess chemotherapy-induced peripheral neuropathy: the QLQ-CIPN20. Eur J Cancer..

[11] Fayers PM, Aaronson NK, Bjordal K (2001). *The EORTC QLQ*-*C30 Scoring Manual*.

[12] Twisk J, de Vente W (2002). Attrition in longitudinal studies: how to deal with missing data. J Clin Epidemiol..

[13] Cohen J (1988). Statistical Power Analysis for the Behavorial Sciences.

[14] Norman GR, Sloan JA, Wyrwich KW (2003). Interpretation of changes in health-related quality of life: the remarkable universality of half a standard deviation. Med Care..

[15] Noordman BJ, Shapiro J, Spaander MC (2015). Accuracy of detecting residual disease after CROSS neoadjuvant chemoradiotherapy for esophageal cancer (preSANO trial): rationale and protocol. JMIR Res Protoc..

[16] Noordman BJ, Spaander MC, Wijnhoven BP, et al. Accuracy of detecting residual disease after neoadjuvant chemoradiotherapy for oesophageal cancer (preSANO trial). *Lancet Oncol.* 2017. (**In press**). 10.1016/S1470-2045(18)30201-8 29861116

[17] Charlson ME, Pompei P, Ales KL, MacKenzie CR (1987). A new method of classifying prognostic comorbidity in longitudinal studies: development and validation. J Chronic Dis..

[18] Cools-Lartigue J, Jones D, Spicer J (2015). Management of dysphagia in esophageal adenocarcinoma patients undergoing neoadjuvant chemotherapy: can invasive tube feeding be avoided?. Ann Surg Oncol..

[19] Ruol A, Rizzetto C, Castoro C (2010). Interval between neoadjuvant chemoradiotherapy and surgery for squamous cell carcinoma of the thoracic esophagus: does delayed surgery have an impact on outcome?. Ann Surg..

[20] Derogar M, Lagergren P (2012). Health-related quality of life among 5-year survivors of esophageal cancer surgery: a prospective population-based study. J Clin Oncol..

[21] Schandl A, Lagergren J, Johar A, Lagergren P (2016). Health-related quality of life 10 years after oesophageal cancer surgery. Eur J Cancer..

[22] Djarv T, Lagergren J, Blazeby JM, Lagergren P (2008). Long-term health-related quality of life following surgery for oesophageal cancer. Br J Surg..

[23] Blum MA, Taketa T, Sudo K, Wadhwa R, Skinner DB, Ajani JA (2016). Chemoradiation for esophageal cancer. Thorac Surg Clin..

[24] Putora PM, Bedenne L, Budach W (2015). Oesophageal cancer: exploring controversies overview of experts’ opinions of Austria, Germany, France, Netherlands, and Switzerland. Radiat Oncol..

