# Supplementary material for: Quality of Life During and After Completion of Neoadjuvant Chemoradiotherapy for Esophageal and Junctional Cancer
Source: Ann Surg Oncol. 2019 Oct 16;26(13):4765–72. doi: 10.1245/s10434-019-07779-w (PMC6864114; doi:10.1245/s10434-019-07779-w)

**Supplementary Table 1.** Overall effects on primary and secondary endpoints of the presence of residual disease during clinical response evaluation (CRE), comorbidities (Charlson comorbidity index, CCI), ASA score (ASA), age, gender, histology and clinical T-stage (cT). Patients who had residual disease during CRE had worse odynaphagia levels, patients with higher CCI experienced more fatigue and patients with higher cT-stage had more weight loss.

| **Status** | **CRE-1** | **ASA** | **CCI** | **Age** | **Gender** | **Histology** | **cT** |
| --- | --- | --- | --- | --- | --- | --- | --- |
| **Primary endpoints** |  |  |  |  |  |  |  |
| Physical functioning | 0.925 | 0.236 | 0.012 | 0.168 | 0.968 | 0.210 | 0.750 |
| Odynophagia | **0.004** | 0.292 | 0.158 | 0.023 | 0.379 | 0.596 | 0.794 |
| Sensory symptoms | 0.796 | 0.402 | 0.096 | 0.134 | 0.823 | 0.699 | 0.958 |
| **Secondary endpoints** |  |  |  |  |  |  |  |
| Global quality of life | 0.675 | 0.706 | 0.006 | 0.062 | 0.533 | 0.961 | 0.761 |
| Fatigue | 0.487 | 0.520 | **<0.001** | 0.066 | 0.710 | 0.177 | 0.960 |
| Weight loss | 0.046 | 0.148 | 0.425 | 0.132 | 0.627 | 0.049 | **0.003** |
| Motor symptoms | 0.150 | 0.014 | 0.166 | 0.571 | 0.244 | 0.924 | 0.249 |

**Supplementary Table 2.** Effects on changes over-time for primary and secondary endpoints of the presence of residual disease during clinical response evaluation (CRE), comorbidities (Charlson comorbidity index, CCI), ASA score (ASA), age, gender, histology and clinical T-stage (cT). Patients who had residual disease during CRE or higher ASA-score had increased weight loss over time (see supplemental figure 1).

| **Status** | **CRE-1** | **ASA** | **CCI** | **Age** | **Gender** | **Histology** | **cT** |
| --- | --- | --- | --- | --- | --- | --- | --- |
| **Primary endpoints** |  |  |  |  |  |  |  |
| Physical functioning | 0.196 | 0.327 | 0.935 | 0.831 | 0.790 | 0.954 | 0.504 |
| Odynophagia | 0.103 | 0.278 | 0.306 | 0.495 | 0.798 | 0.896 | 0.009 |
| Sensory symptoms | 0.565 | 0.661 | 0.559 | 0.372 | 0.031 | 0.591 | 0.689 |
| **Secondary endpoints** |  |  |  |  |  |  |  |
| Global quality of life | 0.014 | 0.011 | 0.080 | 0.202 | 0.055 | 0.207 | 0.050 |
| Fatigue | 0.055 | 0.088 | 0.055 | 0.273 | 0.066 | 0.034 | 0.115 |
| Weight loss | **0.001** | **0.004** | 0.988 | 0.772 | 0.856 | 0.334 | 0.986 |
| Motor symptoms | 0.053 | 0.455 | 0.066 | 0.348 | 0.201 | 0.385 | 0.498 |

**Supplementary Figure 1.** Effects on changes over-time for the presence of residual disease during clinical response evaluation (CRE) and ASA score (ASA). Patients who had residual disease during CRE or higher ASA-score had increased weight loss over time.
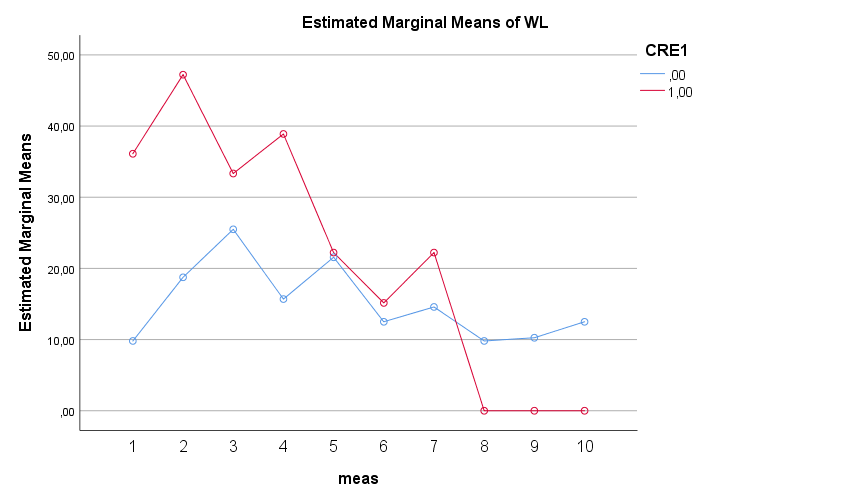


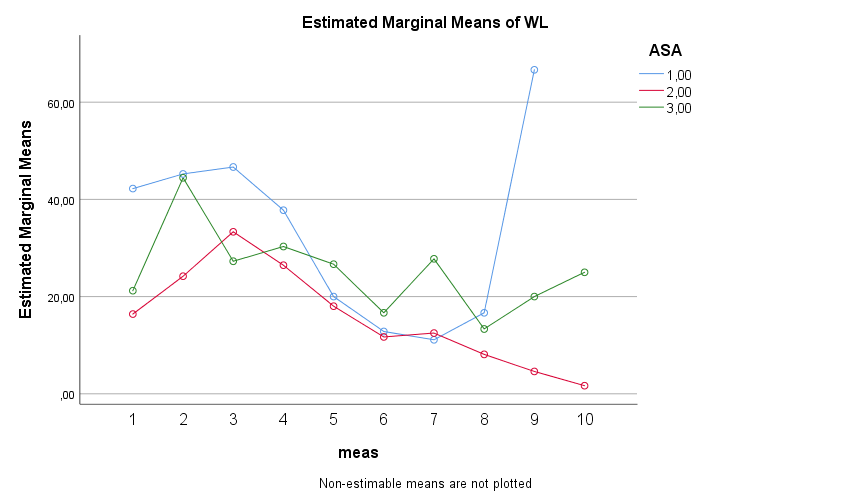

Supplement: Supplementary file 1 — Supplementary material 1 (DOCX 72 kb) [file 10434_2019_7779_MOESM1_ESM.docx]
